# Understanding the Tumor Microenvironmental Mechanisms Driving Immunotherapy Resistance in Colorectal Cancer Liver Metastases

**DOI:** 10.32604/or.2025.074093

**Published:** 2026-03-23

**Authors:** Candela Cives-Losada, Cristiana Soldani, Michela Anna Polidoro, Barbara Franceschini, Ana Lleo, Marcello Di Martino, Matteo Donadon

**Affiliations:** 1 Department of Health Sciences, Università del Piemonte Orientale, Novara, 28100, Italy; 2 Laboratory of Hepatobiliary Immunopathology, IRCCS Humanitas Research Hospital, Rozzano, Milan, 20089, Italy; 3 Department of Biomedical Sciences, Humanitas University, Pieve Emanuele, Milan, 20072, Italy; 4 Division of Internal Medicine and Hepatology, Department of Gastroenterology, IRCCS, Humanitas Research Hospital, Rozzano, Milan, 20089, Italy; 5 Division of General Surgery, University Maggiore Hospital della Carità, Novara, 28100, Italy

**Keywords:** Liver metastasis, tumor microenvironment, cancer, immunotherapy, chemoresistance

## Abstract

Colorectal cancer (CRC) is the second deadliest cancer worldwide, being the presence of metastasis, mainly in the liver, a major contributor to high mortality rates in affected patients. The tumor microenvironment (TME)—comprised of interacting endothelial, stromal, and immune cells—plays a critical role in creating a supportive niche for tumor cell colonization and immune evasion and, thus, the establishment of metastases. The liver’s intrinsic nature further facilitates the development of immune tolerance, mediated by regulatory T cells, myeloid-derived suppressor cells, and soluble factors such as anti-inflammatory cytokines, which together dampen antitumor immune responses. This immunosuppressive milieu contributes significantly to resistance to immune checkpoint inhibitors, limiting the efficacy of immunotherapy in metastatic CRC. Deciphering the complex crosstalk between metastatic CRC cells and TME within the liver is essential for developing novel, effective immunotherapeutic approaches. Several strategies to overcome this lack of response are under research, including combination therapies, novel compounds, and approaches that target TME components. The scope of this review is to synthesize recent advances in the characterization of the hepatic metastatic microenvironment and emerging therapeutic approaches aimed at overcoming immune resistance in CRC liver metastases.

## 1 Introduction

Colorectal cancer (CRC) is the third most common cancer worldwide but the second leading cause of cancer-related death [1]. These tumors are highly invasive, as metastases are sometimes found at the time of diagnosis (around 15%–30% of cases) or are lately developed (20%–50%) [2] most commonly in the liver due to its anatomical position, metabolism, and immune microenvironment, but also in the lungs, peritoneum, and distant lymph nodes [3,4]. The occurrence of liver metastases has a severe impact on the patients’ prognosis and quality of life [3].

Surgery is the standard of care for localized CRC, whereas unresectable metastatic CRC (mCRC) requires systemic or localized therapy for disease control. Among the therapeutic options are chemotherapy based on fluoropyrimidines (mainly 5-fluorouracil) in combination with oxaliplatin (FOLFOX) and/or irinotecan (FOLFIRI), therapies targeting either vascular endothelial growth factor (VEGF) pathway (bevacizumab and ramucirumab) or EGFR (cetuximab and panitumumab) [2,5,6], and immunotherapy (pembrolizumab, ipilimumab, or nivolumab) [2,7].

To select the most suitable treatment, it is recommended to test the molecular profile of the tumor, such as mismatch repair (MMR) and microsatellite instability (MSI) status, and mutations of KRAS, NRAS, and BRAF [8]. Indeed, immunotherapy is effective particularly in a subset of patients with deficient MMR and high MSI (12%–20% of locally advanced but only 5% of metastasized tumors [9]), also characterized by high tumor mutational burden (TMB), the consequent formation of neoantigens leading to immune cell recognition [10]. Thus, pembrolizumab is indicated, both in the U.S.A. and Europe, as first-line treatment in these patients, while the combination of nivolumab and ipilimumab is recommended when the tumor progresses after first-line chemotherapy [2,7,11]. Although inhibiting tumor development by stimulating the appropriate immune response through using immune checkpoint inhibitors (ICIs) is a promising treatment strategy, up to 50% of patients with high MSI present with resistance to ICIs [12], while it has shown no benefit in microsatellite stable (MSS) tumors.

Despite the improvement of the survival rates of patients with mCRC in the last two decades, thanks to the identification of molecular traits and great advances in treatment options, the five-year overall survival (OS) remains low (26%) [13], and relapse is frequent (25%–40%) [14]. This showcases its aggressiveness and highlights the need for a more comprehensive understanding of the mechanisms of resistance to enable the development of novel strategies to overcome them.

In fact, growing attention has been devoted to the role of the tumor microenvironment (TME) in shaping patient responses to both chemotherapy and immunotherapy, given its critical influence on tumor growth and progression [15,16]. TME is very intricate as it comprises diverse cellular components, the extracellular matrix (ECM), as well as cytokines and signaling molecules secreted by both tumoral and surrounding cells [17]. Moreover, the complexity of TME increases at metastasis sites, as their characteristics differ from those of the primary tumor. In particular, the one in the liver is prone to minimizing treatment efficacy due to its thicker ECM, certain vasculature design leading to hypoxic areas, and increased evasion of immune surveillance [18–20].

Taking the relevance of this topic into account, the aim of this review is to summarize the available information on the TME of liver-metastasized CRC, its interaction with tumor cells resulting in resistance to immunotherapy, along with new strategies to resolve such a lack of response.

## 2 Tumor Microenvironment in Colorectal Cancer with Liver Metastasis

**Figure 1:**
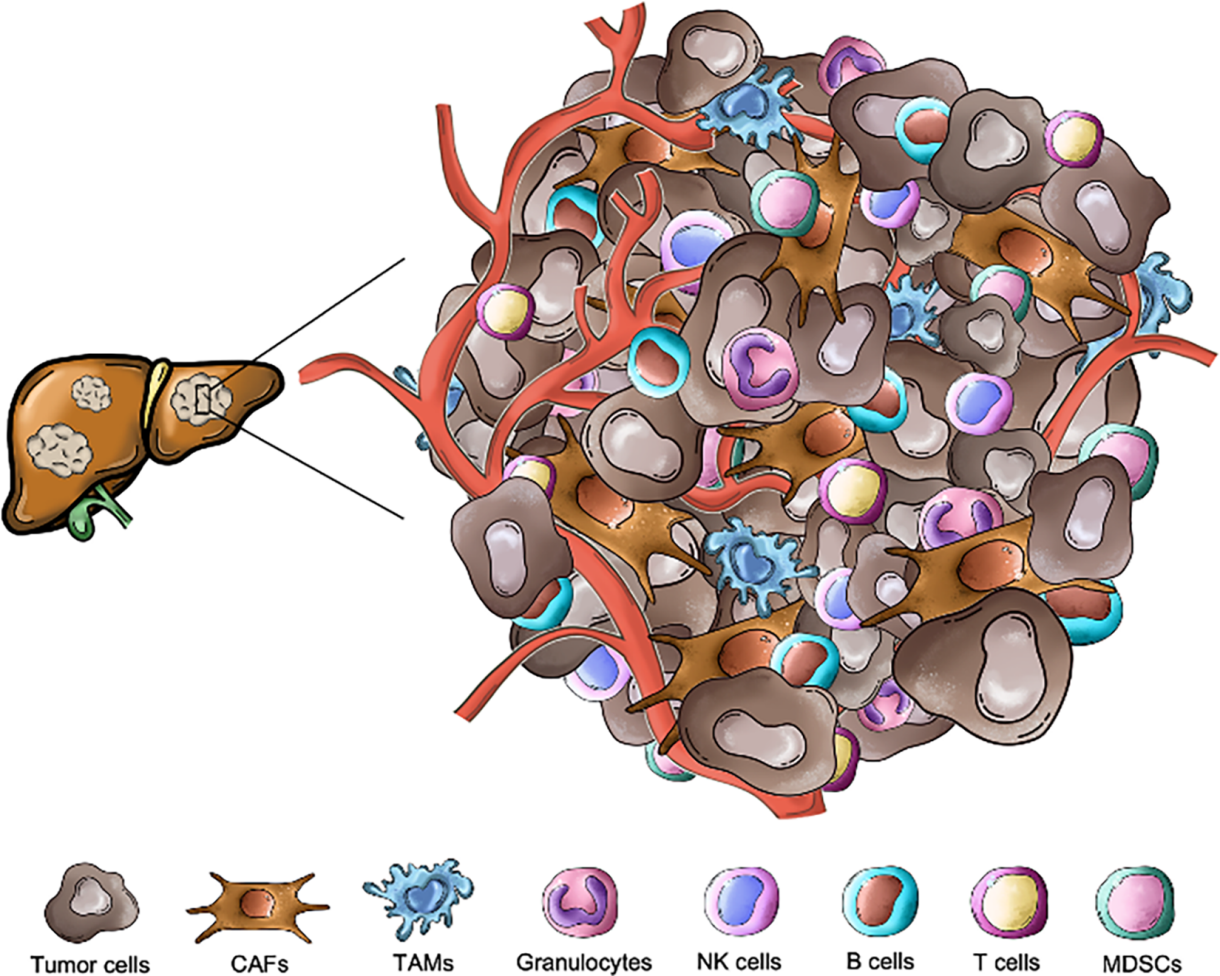
Cellular landscape of the liver metastatic niche in colorectal cancer. The hepatic tumor microenvironment is composed of cancer cells, different immune populations and stromal elements. Key immune subsets include lymphoid cells—T cells, natural killer (NK) cells, B cells and myeloid cells—tumor-associated macrophages (TAMs), myeloid-derived suppressor cells (MDSCs), granulocytes, which exert both pro- and anti-tumor functions. Non-immune stromal components such as cancer-associated fibroblasts (CAFs) and endothelial cells further shape the tolerogenic and immunosuppressive milieu characteristic of colorectal liver metastases. All the pictures present in the figure are original and were hand-drawn

Among the different TME cell components are, besides malignant cells, stromal, endothelial, as well as innate and adaptive immune cells. These cells, embedded in dense and highly structured ECM, communicate both through direct cell-to-cell interactions or by indirect signaling mechanisms (release of soluble factors like cytokines and chemokines) (Fig. 1). This complex crosstalk within the liver metastatic niche supports tumor growth, progression, and immune evasion, which undermines the efficacy of anti-tumor immunity and immunotherapeutic strategies [21,22].

### 2.1 Cell Types within the Liver Tumor Microenvironment

#### 2.1.1 Stromal Cells

Stromal cells are actively involved in remodeling the ECM through the production and secretion of components like collagen and fibronectin. Although cancer-associated fibroblasts (CAFs) are the major stromal population, activated hepatic stellate cells (HSCs) are also important contributors to the creation of pro-tumoral immunosuppressive niche in the liver and a great source of CAFs [23,24]. Moreover, they release growth factors and cytokines, such as, CXCL5, TGFβ, and VEGF which favor inflammation, immune escape and angiogenesis, respectively, shaping tumor growth, invasion and metastasis and, thus, the patient’s prognosis [25–27].

#### 2.1.2 Endothelial Cells

Tumor-associated endothelial cells (ECs) play a key role in angiogenesis, however the formed vasculature within the metastatic tissue is abnormal and “leaky”, which affects the amount of nutrients and oxygen that are delivered to tumor cells, contributing to the formation of hypoxic regions. Not only can ECs constitute a barrier for immune cell trafficking, but they can also modulate their activity or metabolism—specially T cells—by expressing certain molecules (PD-L1, FasL...) and/or enzymes, like indoleamine 2,3-dioxygenase 1 (IDO1) or arginase (ARG1) [28].

#### 2.1.3 Immune Cells

Another key point in TME is the involvement of the immune system, characterized by many different cell populations among tumor-infiltrating lymphocytes (TILs)—i.e., T cells, B cells, NK cells and myeloid-derived cells—e.g., tumor-associated macrophages (TAMs), myeloid-derived suppressor cells (MDSCs), and dendritic cells (DCs) [29].

Tumor-Infiltrating Lymphocytes (TILs)

Within the T cell compartment, multiple functionally distinct subsets can be distinguished. Cytotoxic T lymphocytes (CD8^+^) play a central role in mediating anti-tumor immunity, releasing factors like granzyme B, perforin, and interferon γ (IFN-γ) to eliminate tumor cells [30]. In fact, high infiltration was associated with better prognosis in CRC patients [31]. However, these cells are often exhausted due to adverse conditions like high reactive oxygen species (ROS) and long-term antigen exposure in the TME. This dysfunctional state is marked by reduced cytokine production and high expression of immune checkpoints such as PD-1 [10,32]. T helper cells (Th, CD4^+^) regulate both innate and adaptive immune responses and consist of multiple subsets whose differentiation and function are shaped by the microenvironment cues. Certain subsets support anti-tumor immunity; for example, Th1 cells, which secrete IFN-γ, tumor necrosis factor alpha (TNF-α), and interleukin 2 (IL-2), or cytotoxic CD4^+^ T cells, which can directly eliminate tumor cells expressing MHC class II [33]. In contrast, subsets such as Th9 (IL-9 producers) and Th17 (IL-17A producers) exhibit context-dependent roles: while they can promote tumor progression by enhancing angiogenesis and suppressing immune responses, they may also facilitate tumor rejection by recruiting and activating cytotoxic immune cells. Th22 cells, characterized by IL-22 expression, contribute to an immunosuppressive microenvironment, partly through the induction of IL-10 production [34]. Regulatory T Cells (Tregs)—characterized by expression of FOXP3 and CD25—suppress immune response by releasing inhibitory factors (IL-10, TGF-β and IL-35) and maintaining immune checkpoints, which reduce cytotoxic T cells activity and inhibit dendritic cells maturation [35]. Their accumulation was associated with poor prognosis in many tumor types, including metastatic CRC [22,36].

B cells are known for presenting antigens and producing antibodies, which can help kill malignant cells and, thus, improve patients’ prognosis. However, a subset called regulatory B (Bregs) cells can enhance angiogenesis, reduce cytotoxic T cell activity by expressing PD-L1 and immunosuppressive cytokines such as IL-10 and TGF-β and, thus, foster immune evasion [17,37].

Natural killer (NK) cells are potent cytotoxic effectors and part of the innate immune system. They can eliminate tumor cells regardless of antigen presentation by producing granzyme B and perforin or facilitating programmed cell death through expression of FasL and TNF-related apoptosis-inducing ligand (TRAIL) [38]. Nonetheless, NK cells can present an exhausted phenotype, characterized by PD-1 expression and impaired function [39].

Myeloid-Derived Cells

TAMs are the most abundant infiltrating immune cells in the TME, often accounting for a substantial proportion—up to 50%—of the total cellular content [40]. Macrophages have elevated plasticity and can adopt diverse phenotypes in response to environmental factors. While M1 phenotype favors inflammation and exhibit anti-tumor activity, M2 supports disease progression due to its immunosuppressive nature. They secrete factors like arginine, IL-10, transforming growth factor-beta (TGF-β) or C-C motif chemokine ligand 5 (CCL5) that inhibit cytotoxic T cell activity and recruit Tregs- and promote ECM remodeling and angiogenesis. In the liver TME, TAMs showed different morphology and localization, which has been correlated with patient prognosis, the poorest outcome in those patients with predominant bigger and pro-tumorigenic M2-like [41–43]. Their presence has also correlated with poor prognosis [22,44,45]. In addition, Kupffer cells, the liver-resident macrophages, can support tumor immune evasion by expressing immune checkpoint molecules which suppress CD8^+^ T cell effector function [46]. Highly metastatic CRC cells potentiate the polarization of Kupffer cells toward an M2 phenotype, thereby promoting tumor invasion and metastasis [47]. Moreover, tumor-educated Kupffer cells actively contribute to immune tolerance by facilitating Tregs priming [3] and promoting the expansion of MDSCs through the secretion of IL-10 and TGF-β [48–50].

MDSCs are immature myeloid progenitors, often enriched in the metastatic liver, which foster an immunosuppressive environment that supports tumor cell growth and immune escape [51,52]. They can inhibit T and NK cells’ function by direct contact killing and/or depletion of essential amino acids crucial for their viability and they can promote Tregs cell expansion through releasing factors like arginine, ROS and immunosuppressive cytokines [22,53,54].

DCs are specialized antigen-presenting cells (APCs) that capture, process, and present antigens to T cells, thus playing a central role bridging innate and adaptive immunity, orchestrating effective immune surveillance against cancer [55]. Infiltration of mature DCs within the tumor has been correlated with better prognosis in multiple cancer types including CRC [56], however, their function is frequently impaired within the TME of liver metastases, limiting anti-tumor immunity [52].

Granulocytes compile neutrophils, eosinophils, and basophils. Among them, tumor-associated neutrophils (TANs) have attracted more attention in oncology as they can be polarized in response to environmental factors such as TAMs, acquiring pro or anti-tumor activity and influencing different processes like angiogenesis, matrix remodeling, and invasion through signaling molecules [52]. Higher infiltration of TANs was found in the peritumoral area of CRC tumors with mutated KRAS, in comparison with wild-type KRAS [57].

Mast cells (MCs) trigger inflammatory responses through the release of histamine, proteases, cytokines (IL-6, IL-9, IL-13 and TNF) and chemokines (CXCL8, CCL2 and CCL5) when activated. They can modulate angiogenesis, vascular permeability, ECM remodeling and tumor invasion [22]. Indeed, low levels of MCs were associated with better outcomes in CRC patients through decreased vascularity [11].

### 2.2 Characteristics of the Immune Landscape in Colorectal Cancer with Liver Metastasis

Although genetic heterogeneity is a CRC hallmark, a four-groups consensus molecular subtype (CMS) classification has been developed based on differential gene expressions of tumor and infiltrating cells, thus they also differ in immune TME. CMS1 subtype (14% of cases) is distinguished by hypermutation, high MSI and CpG island methylator phenotype (CIMP). However, the CMS2 “canonical” subtype (37%) and CMS3 “metabolic” subtype (13%) comprise MSS epithelial tumors, but while CMS2 cases are characterized by chromosomal instability, more oncogene copies, with marked WNT and MYC signaling activation, CMS3 are by their metabolic dysregulation and the appearance of KRAS mutations. On the other hand, the CMS4 “mesenchymal” subtype (23% of cases), shows MSS and gene hypermethylation, activation of pathways related to epithelial-mesenchymal transition (EMT) and stemness—including TGF-β—increased stromal invasion, angiogenesis and immunosuppression. Mixed features are shown in 13% cases [58]. Regarding their immune microenvironments, CMS1 tumors have strong immune activation and thus are also known as the “MSI Immune”, with increased T-cell and NK cell infiltration, accompanied by high expression of immune checkpoints. In this context, evaluation of MSI status in both primary CRC tumors and corresponding metastatic sites revealed high concordance in liver ones, although they differed in peritoneal or ovarian metastasis [59]. Alternatively, CMS4 ones are regarded as “immune-inflamed” because, although they also present immune cells infiltration, most are Tregs and Th17 cells and monocyte-derived cells and, which have immunosuppressive functions. Instead, CMS2 and CMS3 are considered as “cold” or “immune desert” tumors, generally PD-L1 negative and with low immune infiltration [60]. Among these subtypes, CMS4 exhibits the worst prognosis, being the TGF-β hyperactivation and the presence of CAFs and ECs important contributors, as they promote invasion, inflammation, angiogenesis and immunosuppression [22,61,62].

The liver’s distinctive anatomical features, including its fenestrated endothelium, and its immune tolerant default state, make it a hospitable niche for metastatic tumor cells’ colonization [3]. Moreover, tumor cell seeding and nutrient delivery are facilitated by the dual blood supply from the portal vein and hepatic artery. Beyond its architecture, the liver inherently presents immunosuppressive microenvironment (evolved to prevent overreaction to gut-derived antigens, to which it is continuously exposed) [63,64] unlike other metastatic sites such as the lung or lymph nodes where immune surveillance is more robust [65]. This immune tolerance is significantly increased in liver metastases, characterized by the presence of Kupffer cells, exhausted CD8^+^ T cells, Tregs, MDSCs, and TAMs, among other factors. Not only does this contribute to tumor progression but also to drug resistance [22,66], which underscores the need for therapeutic strategies that bypass the liver’s tolerogenic features [67,68]. Besides, the amount of immune infiltrating cells can vary across multiple liver metastases within the same patient [69].

Histological growth patterns (HGPs) of CRLM represent a fundamental determinant of TME interactions and strongly influence immunotherapy sensitivity [70]. CRLM mostly display two overarching HGP categories at the interface of the tumor with the surrounding normal liver: desmoplastic (dHGP) and non-desmoplastic, the latter comprising the replacement (rHGP) and, although less common, pushing (pHGP) subtypes [71]. dHGP lesions form a dense fibrotic frame enriched with CAFs and exhibit robust but aberrant neoangiogenesis, characterized by leaky, dysfunctional vessels with fibrin deposition. This angiogenic phenotype is associated with higher infiltration of immune cells (CD45^+^ leukocytes, CD8^+^ T cells, B cells) with an increased CD8^+^/CD4^+^ ratio, reflecting a more permissive immune landscape and explaining the comparatively better outcomes reported for patients with dHGP [72–75]. Conversely, rHGP metastases grow by co-opting pre-existing sinusoidal vessels without inducing angiogenesis, maintaining normal hepatic architecture as tumor cells intercalate between hepatocytes whereas pHGP lesions expand by compressing adjacent hepatocytes. This vessel-co-option process, marked by stromal barriers, creates a highly immunosuppressed and immune-excluded niche contributing to poor responsiveness to both anti-angiogenic and ICI-based therapies [75,76].

To further complicate tumor biology, transcriptomic adaptation phenomena have been described, which refers to metastatic tumors gradually adopting expression profiles to resemble their target tissue (in this case, the liver) while retaining traits of their tissue of origin. In CRC, this transition involved changes in pathways related to ECM remodeling and angiogenesis [77].

## 3 Mechanisms of Immune Resistance

Immunotherapy has emerged as a promising treatment strategy for several solid tumors, including CRC [22]. Its mechanism of action relies on reactivating the patient’s immune system to recognize and eliminate tumor cells by blocking inhibitory pathways that tumors exploit to suppress immune surveillance [78]. Among these pathways, immune checkpoints play a central role. These regulatory molecules, such as programmed cell death protein 1 (PD-1) and cytotoxic T-lymphocyte–associated antigen 4 (CTLA-4), normally function to maintain immune homeostasis and prevent autoimmunity by downregulating T cell activation [12,79]. However, in the TME, these same mechanisms are often hijacked by cancer cells to induce T cell exhaustion and facilitate immune evasion.

PD-1 (CD279), primarily expressed on activated T cells, B cells, and NK cells, is an inhibitory receptor that suppresses T cell proliferation, cytokine secretion, and cytotoxicity upon interaction with its ligands PD-L1 or PD-L2. These ligands are frequently expressed not only by tumor cells but also by immune cells such as macrophages and DCs [80]. In CRC, a higher PD-L1 expression was observed in certain liver metastasis as compared with the primary tumor, this discordance was significantly associated with tumor differentiation [81] and with CD8^+^ lymphocyte infiltration [65]. Such heterogeneity in PD-L1 expression, which was also reported in other metastasized tumor types [82,83], underscores the complexity of immune evasion in metastatic CRC. The immunosuppressive effect of PD-1 signaling is further enhanced in the presence of TGF-β, which promotes the generation of Tregs, further dampening the antitumor immune response [22].

CTLA-4, another key checkpoint receptor, is constitutively expressed on Tregs and is upregulated on activated T cells. It competes with the co-stimulatory receptor CD28 for binding to B7 ligands (CD80 and CD86) on APCs, but with significantly higher affinity. By outcompeting CD28, CTLA-4 inhibits early T cell activation, limits proliferation, and maintains peripheral tolerance [22].

The most established immunotherapeutic agents are immune checkpoint inhibitors (ICIs), designed to block these inhibitory signals and shift the immune response toward an antitumor phenotype [12]. Monoclonal antibodies such as pembrolizumab and nivolumab target PD-1, preventing its interaction with PD-L1/PD-L2 and restoring T cell activity. Similarly, ipilimumab blocks CTLA-4, enhancing T cell priming and reducing Treg-mediated immunosuppression [22].

Despite the success of ICIs in subsets of patients, particularly those with deficient MMR or high MSI tumors—which are characterized by high neoantigen load and increased immunogenicity—most metastatic CRCs are MSS and do not benefit from these therapies [84]. Nevertheless, a significant proportion of patients still present with intrinsic (“innate” or “primary”) resistance, which constitutes an unresolved and complex issue in immunotherapy [85–87] or develop resistance (“acquired”) during treatment [88,89]. Primary resistance often reflects pre-existing features that prevent effective immune activation, such as defects in antigen presentation, absence of effector T-cell infiltration, or constitutive activation of oncogenic pathways. In contrast, acquired resistance typically emerges under therapeutic pressure, through mechanisms including adaptive secondary mutations that restore immune evasion, such as upregulation of alternative inhibitory receptors which diminish T cell activity [90,91]. From a clinical perspective, distinguishing between these resistance phenotypes is essential: intrinsic resistance demands baseline modulation of the TME to enable immune priming, whereas acquired resistance requires sequential therapeutic strategies and dynamic biomarker monitoring to track treatment response [92–95]. Notably, the presence of liver metastases has been associated with a particularly poor response to ICIs, independent of tumor mutational status [19,96].

**Figure 2:**
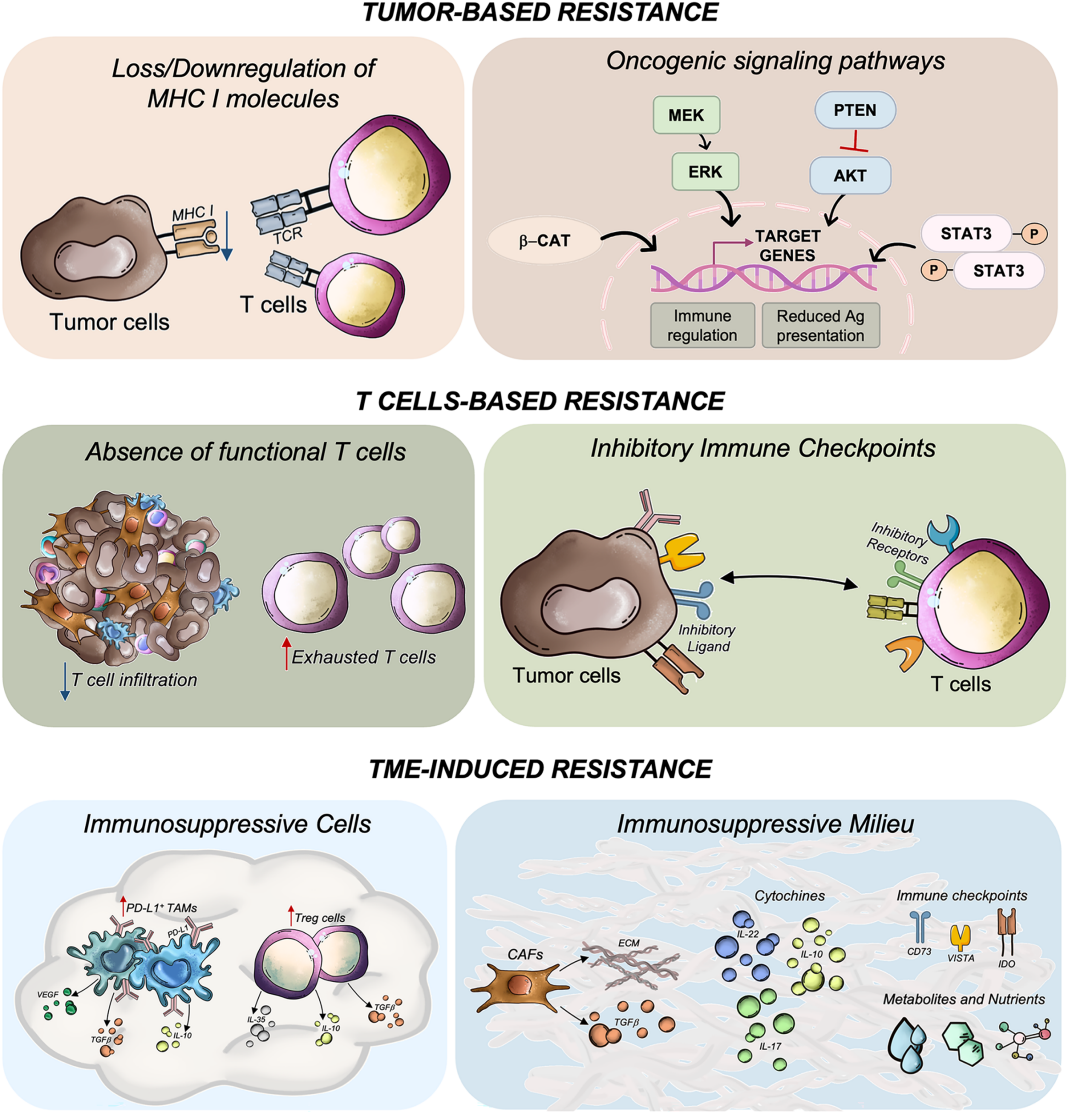
Mechanisms of resistance to immunotherapy in colorectal cancer with liver metastases. Resistance arises from a complex interplay of tumor-intrinsic alterations, dysfunctional T cell responses, and an immunosuppressive tumor microenvironment (TME). Tumor-intrinsic mechanisms include impaired antigen presentation, mutations in oncogenic signaling pathways, and low tumor immunogenicity. T cell-related resistance involves the absence of functional cytotoxic CD8^+^ T cells due to poor tumor infiltration (immune-cold tumors), accumulation of immunosenescent or exhausted T cells, and upregulation of inhibitory immune checkpoints (i.e., PD-1, CTLA-4, LAG-3, TIGIT), which collectively promote immune tolerance. The TME further contributes through elevated levels of immunosuppressive cells, including regulatory T cells (Tregs) and tumor-associated macrophages (TAMs), which secrete inhibitory cytokines such as TGF-β, IL-10, and IL-17. Cancer-associated fibroblasts (CAFs) also play a key role by releasing immunoregulatory factors and remodeling the extracellular matrix, thereby hindering immune cell infiltration. Additionally, the TME is enriched with immunosuppressive metabolites and nutrient-depleting conditions that impair T cell function, reinforce immune evasion and resistance to immune checkpoint inhibitors. Ag, antigen; AKT, AKT serine/threonine kinase 1; β-cat, beta catenin; CD73, cluster of differentiation 73; ECM, extracellular matrix, ERK, extracellular signal-regulated kinase; IDO, indoleamine 2,3-dioxygenase 1; IL, interleukin; MHC, major histocompatibility complex; MEK, mitogen-activated protein kinase kinase; P, phosphorylated; PD-L1, programmed cell death 1 ligand 1; PTEN, phosphatase and tensin homolog; STAT3, signal transducer and activator of transcription 3; TCR, T-cell receptor; TGF-β, transforming growth factor beta; VEGF, vascular endothelial growth factor; VISTA, V-set immunoregulatory receptor. All the pictures present in the figure are original and were hand-drawn

Multiple mechanisms have been proposed to explain the limited efficacy of ICIs in liver metastases, including target heterogeneity, tumor-intrinsic alterations, severe T cell exhaustion, and a complex immunosuppressive landscape [87] (Fig. 2). These mechanisms are further exacerbated by features such as stromal fibrosis, hypoxia, and a pro-inflammatory cytokine milieu that collectively foster immune exclusion and dysfunction in metastatic sites [97].

### 3.1 Tumor-Based Resistance

#### 3.1.1 Absence of Antigenic Proteins and Defective Antigen Presentation

Given the mechanism of action of ICIs, the presence of a sufficiently high TMB along with right antigen presentation is a key determinant of response to PD-1/PD-L1 blockade [98,99].

TMB is typically low (<10 mutations/Mb) in MSS CRC resulting in poor immunogenicity and reduced neoantigen-driven CD8^+^ T cell activation, which contributes to treatment resistance [100].

Moreover, impaired antigen presentation profoundly impacts immune recognition. Loss or downregulation of MHC class I molecules may occur via β_2_-microglobulin (β2m) mutations, epigenetic silencing of HLA genes, or deficiencies in antigen-processing components such as TAP1, tapasin, and LMP2/TAP2. For instance, TAP expression is reduced in KRAS-mutated CRC, which compromises peptide presentation to cytotoxic T lymphocytes [80,101]. β2m loss and MHC-I dysfunction can even occur in high MSI CRC with robust T cell infiltration due to immune selection [101].

In the context of liver metastases, antigen presentation may be further impaired by the tolerogenic hepatic microenvironment, which promotes antigen uptake by Kupffer cells and liver sinusoidal endothelial cells, leading to T cell inactivation [80].

#### 3.1.2 Oncogenic Signaling Pathways

Immune resistance can be also mediated by several oncogenic pathways intrinsic to tumor cells, through suppressing T cell infiltration and regulating PD-L1 expression also affected by inflammatory cues.

Aberrant activation of the WNT/β catenin pathway signaling correlates with a reduced CD8^+^ T cell infiltration in CRC as it decreases the expression of recruiting chemokines such as CCL4 while it increases immune checkpoints like CD47 [102–104]. MAPK pathway activation—secondary to RAS mutations—enhances PD-L1 expression by mRNA stabilization [80]. Moreover, loss of PTEN activates the PI3K/AKT axis, which leads to PD-L1 upregulation and creation of a “cold” TME in multiple tumor types, including CRC [105].

Transcription factors such as STAT3 contribute to the upregulation of PD-L1 on tumor cells, both through intrinsic signaling pathways and in response to external stimuli. Proinflammatory cytokines like IFN-γ and IL-6 enhance this effect via activation of the JAK/STAT1 and STAT3 pathways, respectively. However, defects in IFN-γ signaling, arising from mutations in JAK1/2, IFN-γ receptors, or downstream effectors like IRF1, impair the induction of MHC molecules and antigen presentation machinery, ultimately diminishing the effectiveness of PD-1/PD-L1 blockade [106].

Although over 50% of CRC patients may exhibit tumor PD-L1 positivity (≥10% cutoff), PD-L1 alone remains an unreliable biomarker for predicting ICI response due to challenges such as intratumoral heterogeneity, discordance between primary and metastatic lesions, and a lack of standardized scoring criteria [11,107].

### 3.2 T-Cell-Based Resistance

#### 3.2.1 Absence of Functional T-Cells

The reduced presence or dysfunction of T cells within the TME represents a critical factor influencing immunotherapy response. Even when antigens are present, the immune system’s capacity to reach an effective response is hindered by the limited infiltration or senescence of effector T cells [108]. This is particularly challenging in CRC, as MSS tumors typically exhibit a non-inflamed or “cold” TME characterized by low densities of TILs, especially CD8^+^ cytotoxic T cells, compared to high MSI tumors. Many CD8^+^ T cells in MSS CRC lack CD28 expression, a critical co-stimulatory molecule, suggesting a senescent-like state with reduced proliferative and cytotoxic capabilities [109].

Additionally, in liver metastases, this T cell dysfunction is exacerbated. Clinical and preclinical studies have shown that the hepatic niche is particularly refractory to ICIs due to reduced marginal CD8^+^ T cell infiltration, and accumulation of PD-1^+^ TAMs at the TME of metastases [19]. Notably, metastatic liver disease has been shown to induce systemic deletion or exhaustion of tumor-specific CD8^+^ T cells, impairing immunotherapy efficacy even at distant tumor sites [96,110,111].

#### 3.2.2 Inhibitory Immune Checkpoints

Besides the immune checkpoints targeted with FDA/EMA-approved ICIs, there are other factors that can cause T-cell exhaustion and modify the patients’ immune response. Indeed, PD-1/PD-L1 blockade efficacy can be undermined in MSS CRC tumors when other inhibitory pathways, including CTLA-4, TIM-3, LAG-3, TIGIT, VISTA, and IDO1, are also activated [80]. While LAG-3, expressed on activated T cells, Tregs, and NK cells, suppresses immune activation by binding MHC class II molecules [112], TIGIT, expressed on T cells and NK cells, binds to CD155 and CD112 on tumor cells or APCs and competes with the co-stimulatory receptor CD226, exerting its inhibitory effect on immune activation [113]. These are frequently simultaneously expressed on exhausted CD8^+^ and CD4^+^ T cells, as well as on Tregs, reinforcing a suppressive immune environment. For instance, the presence of LAG-3^+^ FOXP3^+^ Tregs in CRC has been associated with poor clinical outcomes, and the co-expression of PD-1 with CTLA-4 or LAG-3 on CD8^+^ T cells is indicative of deeper functional exhaustion, often refractory to immunotherapy [114,115].

### 3.3 TME-Induced Resistance

#### 3.3.1 Immunosuppressive Cells: TAMs, MDSCs, Tregs

Liver metastases represent a particularly hostile immune environment, characterized by a unique immunosuppressive milieu which often leads to therapeutic resistance even in tumors with immunogenic features such as high MSI status. Among the challenging factors that promote immune evasion are a marked accumulation of PD L1^+^ TAMs, expansion of MDSCs, and enrichment of Tregs, working together to create a profoundly immunosuppressive TME that hampers cytotoxic T cell infiltration and function, thereby limiting the efficacy of PD-1/PD-L1 blockade [104].

TAMs in CRC are predominantly inclined toward an M2-like phenotype, while single-cell studies have identified a particularly enriched population of SPP1^+^ M2-like macrophages, which induce T cell apoptosis through IL-10/STAT3 signaling and Fas/FasL mechanisms and whose presence is associated with poor prognosis [104]. Furthermore, TAMs contribute to immune evasion by secreting immunosuppressive cytokines such as IL-10, TGF-β, and pro-angiogenic factors such as VEGF and by releasing chemokines like MCP 1 and MIP 1α/β that facilitate tumor cell invasion and further immune suppression [80,101,116]. Besides, PD-L1 expression is frequently observed not only in tumor cells but also in surrounding TAMs in liver metastases, contributing to T cell suppression through the PD-1/PD-L1 axis and correlating with resistance to immunotherapy and poorer outcomes [80,117–119]. Interestingly, not only the expression profile but also the morphology of TAMs at the interface of CRC liver metastases correlates with patient survival and has been shown to predict disease recurrence after hepatectomy [42].

MDSCs, particularly polymorphonuclear (PMN-MDSCs) and monocytic (M-MDSCs) subtypes, expand in the metastatic TME, especially under IL 1β–rich conditions [86]. Importantly, this immunosuppressive phenotype is already present in the hepatic pre-metastatic niche and has been clearly associated with tumor progression and invasion. These cells impair T cell function or even deplete them by inducing apoptosis while they also promote Treg differentiation, hindering the antitumor immune response [116,120].

Tregs are highly enriched in CRC compared to healthy tissue and play a critical role in immune evasion. They contribute to immune suppression through various mechanisms. For one, they secrete immunosuppressive cytokines such as IL-10, TGF-β, and IL-35, consume IL-2, depriving effector T cells from this crucial growth factor, and, for another, they can directly induce apoptosis in both APCs and cytotoxic T cells via granzyme B and perforin pathways [116].

#### 3.3.2 Immunosuppressive Cytokines, Metabolites, and Checkpoints in TME

In addition to cellular components, the TME is enriched with soluble factors and metabolites that further hold immune activity back.

Among the most potent of these mediators is TGF-β, secreted by CAFs and TAMs, which promotes Treg induction and drives immune exclusion, particularly in the mesenchymal CMS4 subtype of CRC [104]. IL-10, secreted by both Tregs and TAMs, hampers CD8^+^ T cell activity by inhibiting T cell receptor signaling and downregulating key effector molecules [116]. IL-17A has been shown to contribute to the creation of a protumor environment by promoting MDSC recruitment and PD-L1 upregulation, while IL-17A blockade could restore sensitivity to PD-1 inhibition in preclinical CRC models by enhancing cytotoxic T cell infiltration [121]. Additionally, elevated serum levels of IL-22 have been associated with chemoresistance in CRC patients, suggesting its potential involvement in broader immune escape mechanisms [34]. Moreover, additional immune checkpoints such as IDO, VISTA, and CD73 can be found within the TME [122], which further reinforces immune resistance.

The presence of specific metabolic byproducts within the TME can also suppress T cell function. In addition to competition with tumor and stromal cells for essential nutrients such as glucose, glutamine, and tryptophan, this metabolic stress results in nutrient-deprived or “starved” T cells, leading to an immunometabolic crisis that impairs their survival and effector functions, including altered cytokine production. For instance, dysregulated glutamine metabolism in TME has been correlated with poor immune infiltration, contributing to immune evasion and resistance to ICIs in CRC tumors [123]. Furthermore, accumulation of lactate and kynurenine further dampens T cell activity [116]. Extracellular adenosine, generated by stimulation of CD39- and CD73-expressing Tregs and TAMs, binds to A2A receptors on T cells and suppresses their function [124,125].

## 4 Strategies to Overcome Resistance to Immunotherapy

Given the limited efficacy of ICIs in MSS CRC, which represents most metastatic cases, and the relatively small number of patients currently benefiting from these therapies, multiple strategies are being explored to overcome both primary and acquired resistance [126]. One major line of investigation focuses on combination approaches, administrating ICIs alongside other drugs (like targeted therapies), radiotherapy or oncolytic vaccines with the aim to enhance tumor antigenicity and promote immune activation [127]. In parallel, alternative strategies are targeting specific components of the TME aiming to counteract its immunosuppressive characteristics within the liver metastatic niche. Together, these methods seek to convert “cold” (non-inflamed) CRC metastases into “hot” (inflamed) tumors and restore effective T-cell responses [111,126].

### 4.1 Combination Therapies

**Figure 3:**
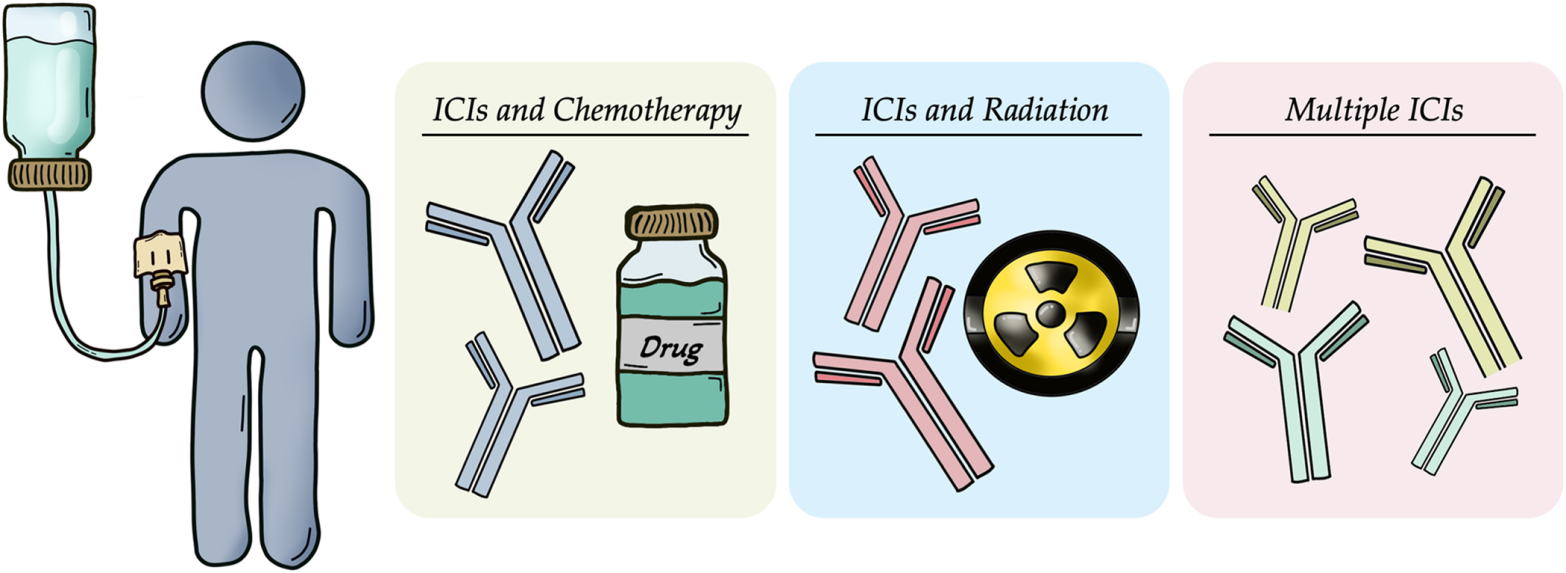
Combination therapies targeting mechanisms of resistance to immunotherapy in colorectal cancer with liver metastases. Approaches include the use of immune checkpoint inhibitors (ICIs) in conjunction with chemotherapeutic agents (cytotoxic and anti-angiogenic drugs), radiotherapy or additional ICIs to enhance antitumor immune responses and overcome immune evasion. All the pictures present in the figure are original and were hand-drawn

Combinatorial regimens are particularly promising in hepatic disease due to the liver’s intrinsic immune tolerance, which often diminishes the effectiveness of ICIs. By simultaneously reducing tumors, remodeling the tumor vasculature, and reactivating immune responses, such multi-target approaches are expected to act synergistically and achieve better therapeutic effects in CRC patients with liver involvement and constitute a promising strategy to overcome ICI resistance [96,111,126,127] (Table 1) (Fig. 3).

**Table 1:** Combination therapies strategies under investigation in liver metastasized colorectal cancer

Agents	Combination strategy	Trial information	Outcomes/Results	References
Nivolumab + Oxaliplatin	ICI (Anti-PD-1) + CC	METIMMOX, Phase II (NCT03388190)	Enhanced responses in MSS mCRC patients	[131]
Anti-PD-L1 + Anlotinib	ICI (Anti-PD-1) + TKI	Retrospective Exploratory Study	Promising outcomes in CRC patients with resistant metastasis	[145]
Atezolizumab + Ramucirumab	ICI (Anti-PD-L1) + Antiangiogenic (Anti-VEGFR2)	Preclinical studies (*in vivo*)	Improved survival rates in animals	[154]
Nivolumab + FOLFOXIRI + Bevacizumab	ICI (Anti-PD-1) + CC + Antiangiogenic (Anti-VEGF)	NIVACOR, Phase II (NCT04072198)	Promising activity in patients with mCRC mutated RAS/BRAF	[155,156]
Nivolumab + mFOLFOX6 + Bevacizumab	ICI (Anti-PD-1) + CC + Antiangiogenic (Anti-VEGF)	CheckMate 9X8, Phase II (NCT03414983)	Higher RR when nivolumab was included compared with chemotherapy alone	[157]
Sintilimab + mFOLFOX6 + Bevacizumab	ICI (Anti-PD-1) + CC + Antiangiogenic (Anti-VEGF)	Clinical trial, Phase II	Favorable DCR and RR for patients with deficient MMR mCRC	[158]
Atezolizumab + FOLFOXIRI + Bevacizumab	ICI (Anti-PD-L1) + CC + Antiangiogenic (Anti-VEGF)	AtezoTRIBE, Phase II (NCT03721653)	Survival benefit in both MSS and MSI-H CRC	[126,134,135]
PDR001 + Dabrafenib + Trametinib	ICI (anti-PD-1) + BRAF/MEK inhibitors	Clinical trial (NCT03668431)	Promising activity in BRAF-mutant CRC	[136,137]
Anti-PD-1 + Trilaciclib or Palbociclib	ICI (Anti-PD-1) + CDK4/6 inhibitors	Preclinical studies (*in vivo*)	Improved response to PD-1 blockade	[138]
Zimberelimab + RT	ICI (Anti-PD-L1) + RT	HaRyPOT, Phase I (NCT06045286)	Enhanced immune response and modified TME	[159]
Envafolimab + RT + CAPEOX	ICI (Anti-PD-L1) + RT + CC	PRECAM, Phase II (NCT05216653)	Favorable efficacy in MSS patients	[126,146]
Botensilimab + Balstilimab	ICIs (Anti-PD-1 + Anti-CTLA-4)	NEST, Phase I/II (NCT05608044)	Durable immunologic responses in MSS CRC	[126,160]
Nivolumab + Ipilimumab (after Temozolomide)	ICIs (Anti-PD-1 + Anti-CTLA-4)	MAYA, Phase II (NCT03832621)	Enduring clinical benefits in in MSS mCRC (with silenced MGMT)	[161]
Nivolumab + Ipilimumab	ICIs (Anti-PD-1 + Anti-CTLA-4)	CheckMate 142, Phase II (NCT02060188)	Enduring clinical benefits in high MSI mCRC	[162]
Pembrolizumab + Favezelimab	ICIs (Anti-PD-1 + Anti-LAG-3)	Clinical trial, early-phase (NCT02720068)	Promising antitumor activity was observed with combination therapy in mCRC	[149,150]
Atezolizumab + Tiragolumab	ICIs (Anti-PD-L1 + Anti-TIGIT)	Clinical trial, early-phase	Promising efficacy in high MSI CRC	[113,149]
Bispecific antibody (PD-L1/CD3) + Regorafenib	ICI (Anti-PD-1-CD3) + TKI	Preclinical studies (*in vivo*)	Enhanced T cell cytotoxicity and anti-tumor activity	[151]
Durvalumab + Tremelimumab + mFOLFOX6	ICIs (Anti-PD-1 + Anti-CTLA-4) + CC	MEDITREME, Phase 1b/2 (NCT03202758)	Promising clinical activity in MSS mCRC	[129]
Nivolumab + Ipilimumab + Regorafenib	ICIs (Anti-PD-1 + Anti-CTLA-4) + TKI	Clinical trial, Phase I (NCT04362839)	Clinical benefit in MSS non-hepatic mCRC	[133]

Note: BRAF, B-raf proto-oncogene, serine/threonine kinase; CC, conventional chemotherapy; CD, cluster of differentiation; CRC, colorectal cancer; CTLA-4, cytotoxic T-lymphocyte associated protein 4; DCR, disease control rate; ICI, immune checkpoint inhibitor; LAG-3, lymphocyte activating 3; mCRC, metastatic colorectal cancer; MGMT, O^6^-methylguanine-DNA methyltransferase; MMR, mismatch repair; MSI, microsatellite instability; MSS, microsatellite stability; PD-1, programmed cell death 1; PD-L1, programmed cell death 1 ligand 1; RR, response rate; TIGIT, T cell immunoreceptor with Ig And ITIM Domains; TKI, tyrosine kinase inhibitor; TME, tumor microenvironment; VEGF, vascular endothelial growth factor.

#### 4.1.1 ICIs and Chemotherapy

Chemotherapy can restore immune surveillance even in immune-depressed tumors by inducing cell death, releasing tumor antigens and danger signals, which enhances DCs activation and T-cell priming [22,128,129]. Indeed, adding ICIs to conventional antitumor drugs (i.e., oxaliplatin) has shown enhanced anti-tumor responses in preclinical models [130] and in the subset of MSS metastasized CRC in randomised METIMMOX phase II trial (NCT03388190) [131]. Similarly, clinical studies have demonstrated the benefit of combining anti-PD-1 therapy with anti-angiogenic such as bevacizumab [132] or tyrosine kinase inhibitors (TKIs) such as regorafenib, as they can normalize tumor vasculature which, in turn, relieves hypoxia and allowing greater T-cell infiltration and a reduction of immunosuppressive populations [126]. Besides, regorafenib has been shown to suppress MDSC and reprogram DCs’ function, sensitizing CRC models to ICIs [126] and CRC patients with advanced MSS CRC without liver metastases in a phase I non-randomized City of Hope Study (NCT04362839) [133]. Notably, the addition of atezolizumab, a PD-L1 inhibitor, to a first-line chemotherapy regimen of FOLFOXIRI with bevacizumab reported survival benefits in both MSS and high MSI CRC in the ongoing AtezoTRIBE trial (NCT03721653), attributed in part to enhanced antigen release and vessel remodeling, allowing greater T-cell infiltration into tumors [126,134,135].

In addition, alternative pathways involved in immune evasion are being investigated as therapeutic targets to improve responses to immune checkpoint inhibitors. For instance, pro-tumorigenic MAPK signaling, commonly activated in CRC, can lead to immune resistance by impairing T cell infiltration and promoting immunosuppressive gene expression. In this context, MEK inhibitors have been used to block MAPK signaling, which in turn preserved CD8^+^ T cell function and sensitized tumors to PD-L1 blockade in preclinical CRC models [136] and in BRAF-mutated CRC patients within a clinical trial (NCT03668431) [137]. Furthermore, the inhibition of cyclin-dependent kinases 4 and 6 (CDK4/6) with small molecules has proven to increase the response to PD-1 blockade by rising tumor infiltration and activation of effector T cells [138]. Besides, the potential benefit of combining ICIs with inhibitors of immunosuppressive signaling pathways, such as IDO, TGF-β or WNT/β-catenin, is also being explored in ongoing trials to potentiate immune responses and achieve better treatment responses [139,140].

Another relevant mechanism is autophagy, a cellular process essential for maintaining homeostasis, which also shapes the tumor immune microenvironment. Modulation of autophagy has been shown to enhance antigen processing and presentation, leading to improved recognition by cytotoxic T cells. Therefore, pharmacological inhibitors or modulators of autophagy have been combined with anti-PD-1/PD-L1 therapy, showing promising results in both *in vitro* and *in vivo* tumor models, including enhanced CD8^+^ T cell infiltration and tumor regression [141,142].

#### 4.1.2 ICIs and Radiation

Liver-directed radiotherapy (RT) can reshape the immunosuppressive liver milieu. Thus, RT combination with ICIs has emerged as a promising strategy to enhance treatment efficacy in liver metastasized CRC [143]. In animal models, liver RT restored systemic antitumor immunity by depleting immunosuppressive TAMs and promoting T cell survival and infiltration when combined with ICIs [111].

The triple combination of RT, anti-PD-1 and anlotinib (anti-angiogenic TKI) was tested *in vivo*, using a mouse liver metastasis tumor model, to achieve a synergistic effect by acting on different targets: locoregional therapies induce cell death and antigen release, checkpoint blockade reactivates T-cell effector functions and anti-angiogenic agents normalize the vasculature. A significantly higher infiltration of CD4^+^, CD8^+^ T cells and less MDSCs were observed in treated mice, which translated into improved disease control. Together, this combination remodeled the liver TME from immunologically “cold” to “hot,” thereby enabling effective antitumor immune responses [144]. Promising results were also observed when combining anlotinib and PD-1 blockers in patients with resistant CRC metastasis [145].

Another triple combination—envafolimab (anti-PD-L1), with short-course RT and CAPEOX-based chemotherapy—was tested on locally advanced rectal cancer within the PRECAM trial (NCT05216653), which resulted in a pathologic complete response rate of 62.5% among MSS patients [126,146].

#### 4.1.3 Blocking Multiple Immune Checkpoints

Targeting more than one immune checkpoint at once could help overcome T-cell exhaustion (i.e., combining anti-PD-1 + anti-CTLA-4 agents) [80]. In the NEST trial (NCT05608044), botensilimab (anti–CTLA-4) and balstilimab (anti–PD-1) achieved durable immunologic responses when used as a neoadjuvant regimen in patients with localized MSS CRC [126]. Furthermore, dual immune checkpoint blockade with durvalumab and tremelimumab (anti-PD-L1 and anti-CTLA-4, respectively) was tested in combination with mFOLFOX6 chemotherapy within the single-arm MEDITREME phase 1b/2 trial (NCT03202758), demonstrating high disease control in RAS-mutated MSS metastatic CRC [129].

Emerging immune checkpoints upregulated in dysfunctional T cells within the TME such as LAG-3, TIGIT, TIM-3, and VISTA are being explored in combination with PD-1/PD-L1 inhibitors aiming to restore effector function and broaden the therapeutic response, particularly in MSS and liver-metastatic CRC, where single-agent immunotherapy has limited efficacy [147–149]. For example, dual PD-1/LAG-3 blockade (pembrolizumab plus favezelimab) has shown synergistic effects by reaching modest clinical responses in MSS CRC within an early-phase clinical trial [149,150]. Similarly, tiragolumab (anti-TIGIT) in combination with atezolizumab (anti-PD-L1) have demonstrated promising efficacy in solid tumors, including high MSI CRC [113,149].

Beyond ICIs’ combination, bispecific antibodies (BsAbs), which bind two different antigens or epitopes, represent an innovative strategy to simultaneously engage immune cells and tumor targets. A PD-L1/CD3 BsAb was designed to activate CD3 on T cells while helping them link to PD-L1^+^ tumor cells and demonstrating potent antitumor activity in preclinical CRC models. This effect was further enhanced when combined with regorafenib [151,152].

Although combining ICIs with other agents had shown promising response rates, it is also associated with higher frequency and severity of immune-related toxicities compared to single-agent treatments. Therefore, careful patient selection and close monitoring of side effects are critical when implementing these strategies [96,153].

### 4.2 Targeting the TME

Directly remodeling the TME offers a promising opportunity to improve the efficacy of immunotherapy in liver metastatic CRC, where immunosuppressive signals and structural barriers enable tumors to evade immune surveillance and limit response to current treatments. Among these emerging approaches are enhancing antigen presentation, targeting stromal elements, suppressive cytokines, and immunoregulatory cell populations to disrupt this pro-tumor niche [126] (Table 2).

**Table 2:** Strategies targeting the tumor microenvironment to treat liver metastasized colorectal cancer

Strategy	Target	Mechanism	Expected effect	Evidence	References
	Tumor cells	MHC-I restoration, epigenetic modulation	Enhanced tumor visibility to immune system	Preclinical	[101,116]
Antigen presentation enhancement	DCs	Neoantigen vaccines, Oncolytic viruses	Potentiate immune activation: boost DCs and priming of effector T cells	Preclinical	[126,152]
	DCs	*Ex vivo* DC expansion, GM-CSF activation		Preclinical and early-phase trials	[163]
Overcome physical/biochemical immune exclusion	ECM: filaments	Collagenase, Nanoparticles	Facilitate immune infiltration	Preclinical	[166]
	ECM: soluble factors	Cytokine inhibition (IL-17A/IL-1β blockade)	Reduce MDSC recruitment, boost CD8^+^ T cells	Preclinical	[80,86]
	ECM: metabolic products	Hypoxia modulation	Increase ROS to overcome immunosuppression	Preclinical	[166]
	CAFs or HSCs	anti-FAP, RLN overexpression	Fibroblast inactivation, enhanced T cell infiltration into metastases	Preclinical (RLN), Phase I (anti-FAP antibody)	[22,165]
Potentiate immune-mediated cytotoxicity	T cells	CAR-T cells (e.g., CDH17-targeted)	Direct tumor cell killing by engineered T cells that targeting CRC-specific molecules	Preclinical	[167]
	NK cells	α-GalCer, vaccines	Enhance innate-like immunity in “cold” tumors	Preclinical	[163]
Reprogramming suppressive immune cells	TAMs	CSF1R or CCR5 blockade	Reprogramming to M1 phenotype, improved inflammation	Preclinical and Phase I (CCR5 antagonist)	[117,174]
	MDSCs	Inhibition of PI3Kγ, CCR2/5, CXCR2	Modulating or depleting MDSCs	Preclinical studies	[54,175]
	Tregs	TLR agonists, anti-CD25, cyclophosphamide, IDO inhibitors	Treg depletion or functional inhibition	Preclinical/early clinical	[117]

Note: CAF, cancer-associated fibroblast; CAR-T cell, Chimeric Antigen Receptor T-cell; CCR, C-C motif chemokine receptor; CXCR, C-X-C motif chemokine receptor; DC, dendritic cells; ECM, extracellular matrix; FAP, fibroblast activation protein alpha; GM-CSF, granulocyte-macrophage colony stimulating factor; HSC, hepatic stellate cells; IDO, indoleamine 2,3-dioxygenase 1; IL, interleukin; MDSC, myeloid-derived suppressor cells; MHC; major histocompatibility complex; NK, natural killer; RLN, relaxin; ROS, reactive oxygen species; TAM, tumor-associated macrophage.

#### 4.2.1 Enhancing Antigen Presentation

Different therapies to improve antigen presentation and increase tumor “visibility” to the immune system are under investigation, such as restoring MHC-I expression through epigenetic modulation or stimulating antigen-presenting cells [101,116].

Neoantigen-based vaccines could potentially boost tumor recognition and improve survival for patients in immune-cold subtypes. Thus, cancer vaccines and oncolytic viruses are under development to stimulate endogenous DC activation and boost T cell priming [126]. For instance, machine learning was used to identify tumor antigens and immune targets and tailored to immune subtype-specific responses for CRC patients [152].

Other strategies to boost DC function and overcome tolerogenic signaling include *in vivo* DC activation using GM-CSF or their *ex vivo* expansion, maturation and reinfusion in the patient [163].

#### 4.2.2 Targeting Matrix and Stromal Factors

The ECM and stromal cells in liver metastases can physically and biochemically exclude immune cells. Hence, strategies that interfere with stromal barriers, such as targeting CAFs or HSCs, or that modulate ECM components (i.e., collagenases), could facilitate T cell infiltration and improve therapeutic efficacy [164]. Interestingly, inhibiting activated HSCs through up-regulating the expression of the relaxin (RLN) resolved liver fibrosis and had anti-metastasis effect with PD-L1 in liver metastasized CRC mouse models [165]. However, a human monoclonal anti-fibroblast activation protein (anti-FAP) antibody showed no clinical activity in CRC patients with liver metastasis within a phase I trial [22].

Hypoxia, common in liver metastases, also drives immunosuppression; novel methods such as manganese-based nanoparticles combined with small-molecule modulators have been proposed to locally increase ROS and provide antitumor treatment and improve the efficacy of immunotherapy [166].

Tumor-derived and stromal cytokines can recruit immunosuppressive cells. Therefore, inhibiting pro-tumor cytokine is a strategy to tilt the balance toward immunity. For instance, preclinical work shows that inhibiting IL-17A or IL-1β can blunt MDSC recruitment and reinvigorate CD8^+^ T cells in CRC models [80,86].

#### 4.2.3 Activating Innate-Like Lymphocytes

NK cells could be especially useful in immunologically “cold” tumors like MSS CRC due to their ability to recognize “dangerous” cells independently of MHCs and secrete cytokines—such as IFN-γ, IL-4, and TNF-α—which promote cytotoxicity, enhance DC function, and help recruit CD8^+^ T cells. Strategies using NK cell agonists, α-GalCer–loaded DCs or synthetic glycolipid vaccines are being investigated to activate both NK and CD8^+^ T cells and synergize with DC-based therapies [163].

Adoptive cell therapies, particularly CAR-T cell approaches, are being adapted to solid tumors like CRC to bypass immune evasion mechanisms and directly engage tumor-specific antigens. In liver metastases of CRC, preclinical studies using CAR-T cells targeting cadherin-17 (CDH17), a cell adhesion molecule highly expressed in CRC but largely absent from normal tissue, have shown potent antitumor activity. Systemically administered CDH17-targeted CAR-T cells significantly reduced tumor burden in humanized mouse models [167]. However, translating CAR-T therapy into CRLM faces unique challenges imposed by the tolerogenic hepatic environment. Liver sinusoids can trap and deactivate CAR-T cells through phagocytosis (mediated by TAMs or Kupffer cell) and immunosuppressive molecules such as IL-10, IDO and adenosine. Moreover, antigen heterogeneity between primary and metastatic lesions predisposes to antigen escape following treatment [168,169]. To overcome these barriers, multiple optimization strategies are under investigation. These include regional delivery approaches, such as hepatic artery infusion to enhance intratumoral CAR-T accumulation; dual-target CAR constructs designed to reduce antigen-escape variants [170]; and “armored” CAR-T cells engineered to resist or remodel the suppressive niche, for example by incorporating TGF-β-resistant signaling domains [171] or by secreting IL-7 and CCL19 to improve local T-cell recruitment and survival [172,173].

#### 4.2.4 Reprogramming Suppressive Immune Cells

Targeting specific immunosuppressive cell populations within TME represents another promising strategy to enhance the efficacy of immune ICIs in metastatic CRC.

Agents such as CSF1R inhibitors can deplete TAMs or reprogram them toward a pro-inflammatory M1 phenotype, restoring antitumor cytokine production such as IFNγ. Additionally, CCR5 blockade, which disrupts the CCL5-CCR5 axis active in both tumor and immune cells, has shown potential to reverse pro-metastatic inflammation and repolarize macrophages in patient-derived CRC models. These findings were supported by early-phase clinical trials demonstrating clinical responses in patients with advanced CRC liver metastases [117,174].

Furthermore, approaches aimed at limiting MDSC recruitment (via CCR2/CCR5 or CXCR2 inhibitors), reprogramming their suppressive phenotype (e.g., with PI3Kγ inhibitors), or depleting them altogether have shown encouraging results in preclinical models [54,175].

Therapeutic strategies to reduce Treg activity or rebalance the Treg/Teffector ratio include TLR agonists, anti-CD25 antibodies, cyclophosphamide, and IDO inhibitors [117].

## 5 Future Perspectives

Despite the promising results of ICIs in the subset of tumors that harbor deficient MMR or high MSI, most CRC patients remain unresponsive to available immunotherapy. Unfortunately, the molecular bases of tumor refractoriness remain poorly understood. This poses a major challenge in advancing immune-based treatments for CRC and has encouraged relevant scientific efforts towards gaining knowledge about said resistance, searching for robust predictive biomarkers (besides MSI status), and developing novel treatment strategies able to overcome it.

Characterizing immune and stromal cell populations present in the TME, and their interactions with tumor cells before and after treatment, can shed some light on how different immunotherapies reshape the immune TME and the counteracting mechanisms of resistance. High-throughput platforms such as transcriptomics, proteomics, and metabolomics can dissect the cellular and molecular composition of the TME, providing insights into immune cell states, stromal dynamics, and signaling pathways. Multiplex methodologies, including multiplex immunohistochemistry/immunofluorescence (mIHC/mIF) and multiplexed imaging, further enhance this analytical capacity by simultaneously detecting and mapping multiple proteins within a single tissue sample. These approaches enable detailed visualization of tumor-immune–stromal interactions, facilitating biomarker discovery, identification of novel drug targets, and improved patient stratification [176–178]. Integrating such complex datasets with artificial intelligence and machine learning holds promise for generating a more comprehensive understanding of the tumor immune landscape and ultimately allowing more precise patient stratification.

The identification of reliable biomarkers that can accurately predict patient response to therapy could significantly enhance clinical decision-making and guide personalized treatment strategies, but remains a challenge in CRC. While TMB has been proposed as a potential predictive marker, its clinical utility is still uncertain. Notably, stromal signatures and cytokine profiles have gained attention due to their role in shaping the immune TME. Circulating tumor DNA (ctDNA) has emerged as a powerful dynamic biomarker in CRLM, offering a non-invasive approach to detect minimal residual disease earlier than conventional imaging, predict recurrence after resection, and quantify treatment response—critical for anticipating timely intervention [179–181]. In addition, ctDNA mutational profiling can reveal newly acquired resistance mechanisms, thereby guiding clinical decision-making and informing alternative therapeutic strategies [182]. In parallel, microRNAs have emerged as promising biomarkers of immune resistance, with specific expression patterns correlating with immune activity and treatment outcomes. Importantly, miRNAs can be detected through liquid biopsy, offering a non-invasive and dynamic approach to monitor the tumor’s immune status in real time. This holds great potential not only for early prediction of immunotherapy response but also for tracking resistance mechanisms during treatment.

Several strategies to overcome the limited response to immunotherapy are currently being investigated. Among them are combination therapies, novel compounds, and approaches specifically designed to target key components of the TME, some being evaluated in clinical trials. A particularly promising path involves multi-targeted approaches that combine ICIs with other therapeutic agents such as chemotherapy, anti-angiogenic agents, radiotherapy, or next-generation immune modulators. These combination strategies are designed to reduce the immunosuppressive milieu, increase tumor immunogenicity, for instance, by facilitating more effective antigen presentation, and enhance the recruitment and activation of effector immune cells. Importantly, the heterogeneous immune landscapes across CMS of CRC suggest that therapeutic approaches should be tailored to subtype-specific biology. CMS1 tumors, characterized by MSI and strong immune activation, are most responsive to ICIs, and combination regimens such as ICI plus anti-angiogenic agents are under investigation. In contrast, CMS4 ones exhibit a fibro-inflammatory milieu dominated by TGF-β signaling and stromal exclusion, where rational strategies include TGF-β blockade and CAF-directed therapies. CMS2 and CMS3 subtypes, generally considered “cold” tumors, may require tumor-intrinsic targeted interventions (for example, EGFR blockade) prior to immune reactivation [29,58,183,184].

Across CRLM-specific trials, meaningful cross-study comparison remains difficult due to heterogeneity in design, patient populations, and endpoints; however, several consistent patterns can be identified. ICI-based combinations with targeted agents yield occasional responses, suggesting partial reversal of hepatic immune tolerance. BsAbss show early clinical activity but are limited by cytokine-associated and hepatotoxic effects. Adoptive cell therapies remain feasible yet face unique barriers in CRLM, including profound immunosuppression within the hepatic niche and risks of off-tumor toxicity. As these trials progress, they hold the potential to broaden the therapeutic reach of immunotherapy beyond the currently responsive deficient MMR/MSI-high subgroup, moving toward more personalized and effective treatment strategies for a larger proportion of CRC patients. Together, these observations underscore the importance of biomarker-guided patient selection and more homogeneous trial frameworks to enable robust evaluation of emerging CRLM-specific immunotherapies.

Despite encouraging preclinical results, the clinical translation of many TME-targeted strategies is still limited in CRLM. Most agents were developed for extrahepatic tumor biology and do not adequately address the liver’s intrinsic immune tolerance. Additionally, spatial heterogeneity between primary CRC tumors and liver metastases complicates biomarker interpretation, and resistance is dynamic and frequently multilayered [185]. These barriers highlight the need for rational, sequential, mechanism-based therapeutic combinations, as moving from experimental models to clinically effective interventions remains one of the central challenges in translational oncology.

In conclusion, overcoming immunotherapy resistance in CRC requires a multifaceted approach that integrates multiple innovative techniques. By increasing our understanding of the immune landscape evolution, personalized immunotherapy to achieve the best possible response rates becomes more reachable.

## Data Availability

Not applicable.

## Abbreviations

APC: Antigen-presenting cell
CAF: Cancer-associated fibroblast
CMS: Consensus molecular subtype
CRC: Colorectal cancer
DC: Dendritic cells
ECM: Extracellular matrix
FOLFIRI: Fluoropyrimidines in combination with irinotecan
FOLFOX: Fluoropyrimidines in combination with oxaliplatin
HSC: Hepatic stellate cells
ICI: Immune checkpoint inhibitor
IDO: Indoleamine 2,3-dioxygenase 1
IL: Interleukin
mCRC: Metastatic colorectal cancer
MDSC: Myeloid-derived suppressor cells
MHC: Major histocompatibility complex
MMR: Mismatch repair
MSI: Microsatellite instability
MSS: Microsatellite stable
NK: Natural killer
ROS: Reactive oxygen species
TAM: Tumor-associated macrophage
TILs: Tumor-infiltrating lymphocyte
TKI: Tyrosine kinase inhibitor
TMB: Tumor mutational burden
TME: Tumor microenvironment
Treg: Regulatory T cell
VEGF: Vascular endothelial growth factor
